# Tissue Engineering Whole Bones Through Endochondral Ossification: Regenerating the Distal Phalanx

**DOI:** 10.1089/biores.2015.0014

**Published:** 2015-04-01

**Authors:** Eamon J. Sheehy, Tariq Mesallati, Lara Kelly, Tatiana Vinardell, Conor T. Buckley, Daniel J. Kelly

**Affiliations:** ^1^ Trinity Centre for Bioengineering, Trinity Biomedical Sciences Institute, Trinity College Dublin, Dublin, Ireland.; ^2^ Department of Mechanical and Manufacturing Engineering, School of Engineering, Trinity College Dublin, Dublin, Ireland.; ^3^ School of Agriculture and Food Science, University College Dublin, Belfield, Dublin, Ireland.; ^4^ Department of Anatomy, Royal College of Surgeons in Ireland, Dublin, Ireland.; ^5^ Advanced Materials and Bioengineering Research Centre (AMBER), Royal College of Surgeons in Ireland and Trinity College Dublin, Dublin, Ireland.

**Keywords:** anatomical, biomaterials, stem cells, tissue engineering, endochondral, alginate

## Abstract

Novel strategies are urgently required to facilitate regeneration of entire bones lost due to trauma or disease. In this study, we present a novel framework for the regeneration of whole bones by tissue engineering anatomically shaped hypertrophic cartilaginous grafts *in vitro* that subsequently drive endochondral bone formation *in vivo.* To realize this, we first fabricated molds from digitized images to generate mesenchymal stem cell-laden alginate hydrogels in the shape of different bones (the temporomandibular joint [TMJ] condyle and the distal phalanx). These constructs could be stimulated *in vitro* to generate anatomically shaped hypertrophic cartilaginous tissues that had begun to calcify around their periphery. Constructs were then formed into the shape of the distal phalanx to create the hypertrophic precursor of the osseous component of an engineered long bone. A layer of cartilage engineered through self-assembly of chondrocytes served as the articular surface of these constructs. Following chondrogenic priming and subcutaneous implantation, the hypertrophic phase of the engineered phalanx underwent endochondral ossification, leading to the generation of a vascularized bone integrated with a covering layer of stable articular cartilage. Furthermore, spatial bone deposition within the construct could be modulated by altering the architecture of the osseous component before implantation. These findings open up new horizons to whole limb regeneration by recapitulating key aspects of normal bone development.

## Introduction

A number of clinical situations exist where bone regeneration is required in large quantities, such as for the reconstruction of large bone defects caused by trauma, infection, and skeletal abnormalities, or in circumstances where the regenerative process is compromised, such as in avascular necrosis and atrophic nonunions.^1^ Tissue engineering involves using a combination of cells, three-dimensional (3D) scaffolds, and signaling molecules to repair or regenerate such damaged or diseased tissues.^2,3^ Since no biological therapies exist for whole bone regeneration, tissue-engineered anatomically shaped bone grafts have been proposed as functional replacements for bones lost due to trauma or disease.^4–12^ The approaches adopted in these studies have varied from the selective placement of periosteum, chondrocytes, and tenocytes into a biodegradable synthetic polymer scaffold^4^ to the use of anatomically shaped scaffolds generated from decellularized trabecular bone that were seeded with mesenchymal stem cells (MSCs) and maintained in a flow perfusion bioreactor.^11^ To date, cell-based bone tissue engineering strategies have generally focused on the direct osteogenic priming of MSC-seeded scaffolds in a process resembling intramembranous ossification.^13^ This approach, however, has been hampered by insufficient vascularization of the graft following *in vivo* implantation, thus preventing the necessary delivery of oxygen and nutrients required to ensure cell survival.^14^ The development of a necrotic core within such grafts is a significant challenge in the field of bone tissue engineering,^15^ and one which will be exacerbated by the scaling up of grafts to regenerate whole bones and joints.

The long bones of the body form not by intramembranous, but by endochondral, ossification, whereby chondrocytes in the developing cartilaginous template undergo hypertrophy and direct remodeling of the cartilage into bone.^16^ Cells progressing down the endochondral route are programmed to survive low-oxygen conditions,^17^ such as those experienced by tissue-engineered grafts upon implantation. Furthermore, cells undergoing hypertrophy release proangiogenic factors such as vascular endothelial growth factor for the conversion of avascular tissue to vascularized tissue.^18^ Chondrogenically primed MSCs have been shown to possess an inherent hypertrophic capacity,^19^ leading to an increased interest in the engineering of hypertrophic cartilaginous grafts for bone regeneration.^17,20–27^ This endochondral approach has also been leveraged to engineer osteochondral tissues by spatially regulating endochondral ossification within chondrogenically primed constructs.^28^ These advances pave the way for the engineering of scaled-up anatomically shaped grafts, equipped with functional articular surfaces, for the potential regeneration of whole joints and bones.

The objective of this study was to tissue engineer hypertrophic cartilaginous constructs in the shape of specific bones *in vitro*, which, we hypothesized, would provide a template for the development of an entire bone *in vivo* by recapitulating the process of endochondral ossification. To realize this goal, we fabricated molds from digitized images to generate MSC-laden alginate hydrogels in the shape of various bones. To test our hypothesis, MSC-seeded hydrogels were cast into the shape of the distal phalanx to form the hypertrophic precursor of the osseous component of an engineered long bone. A layer of hyaline cartilage engineered through self-assembly of chondrocytes served as the articular surface of these constructs. The capacity of these anatomically shaped constructs to generate a functional bone was then evaluated by subcutaneous implantation of the engineered phalanx into nude mice following chondrogenic priming *in vitro*.

## Materials and Methods

### Cell isolation and expansion

Bone marrow-derived MSCs were isolated from the femoral shafts of 4-month-old pigs and expanded according to a modified method for human MSCs^29^ in high-glucose Dulbecco's modified Eagle's medium GlutaMAX (hgDMEM) supplemented with 10% v/v fetal bovine serum (FBS), 100 U/mL penicillin–100 μg/mL streptomycin (all Gibco; Biosciences), and 2.5 μg/mL amphotericin B (Sigma-Aldrich) at 20% pO_2_. Following colony formation, MSCs were trypsinized, counted, seeded at density of 5×10^3^ cells/cm^2^ in 500-cm^2^ triple flasks (Thermo Fisher Scientific), supplemented with hgDMEM, 10% v/v FBS, 100 U/mL penicillin–100 μg/mL streptomycin, 2.5 μg/mL amphotericin B, and 5 ng/mL human fibroblastic growth factor-2 (FGF-2; Prospec-Tany TechnoGene Ltd.), and expanded to passage 2. At the end of passage 2, MSCs were frozen in 90% v/v FBS and 10% dimethyl sulfoxide (Sigma-Aldrich) and stored in liquid nitrogen. Porcine chondrocytes were also isolated from the articular cartilage of the femoropatellar joints. Cartilage slices were rinsed with Dulbecco's phosphate-buffered saline (PBS; Sigma-Aldrich) supplemented with 100 μg/mL streptomycin and 2.5 μg/mL amphotericin B and digested with hgDMEM containing collagenase type II (350 U/mL) (Worthington; Langanbach Services) for 12–14 h under constant rotation at 37°C. The resulting cell suspension was filtered through a 40-μm-pore-size cell sieve (Fisher Scientific), centrifuged, rinsed with PBS, and counted using a hemacytometer. Chondrocytes were then frozen in 90% v/v FBS and 10% dimethyl sulfoxide (Sigma-Aldrich) and stored in liquid nitrogen. Before fabrication of anatomically shaped constructs (details below), MSCs and chondrocytes were thawed and expanded for one additional passage (i.e., MSCs to passage 3, chondrocytes to passage 1).

### Chondrocyte self-assembly

Chondrocytes were suspended in hgDMEM supplemented with 10% v/v FBS, 100 U/mL penicillin–100 μg/mL streptomycin, and 2.5 μg/mL amphotericin B at a density of 100×10^6^ cells/mL. Forty microliters of this cell suspension was pipetted into 4% agarose cylindrical wells (Ø5×3 mm), to give a final concentration of 4×10^6^ cells/construct, and allowed to self-assemble for 12 h. Thereafter, constructs were cultured in a chondrogenic medium (CM) consisting of hgDMEM GlutaMAX supplemented with 100 U/mL penicillin/streptomycin (both Gibco), 100 μg/mL sodium pyruvate, 40 μg/mL l-proline, 50 μg/mL l-ascorbic acid-2-phosphate, 4.7 μg/mL linoleic acid, 1.5 mg/mL bovine serum albumin, 1× insulin–transferrin–selenium, 100 nM dexamethasone (all from Sigma-Aldrich), 2.5 μg/mL amphotericin B, and 10 ng/mL of human transforming growth factor-β3 (TGF-β3) (Prospec-Tany TechnoGene Ltd.) at 20% pO_2_ for a period of 4 weeks to form the chondral layer of the tissue-engineered phalanx.

### Fabrication and culture of tissue-engineered alginate phalanx constructs

The distal phalanx of a human skeleton model was scanned using a PICZA 3D Laser Scanner model LPX-250. 3D computer-aided design software was used to render the scans, and the designs were rapid prototyped using the Stratasys Dimension Fused Deposition Modeler to produce a two-part acrylonitrile–butadiene–styrene (ABS) reverse mold. The two-part ABS mold was infiltrated with a 4% agarose/50 mM CaCl_2_ solution and allowed to set. The resulting two-part 4% agarose/50 mM CaCl_2_ mold was assembled and injected with MSC-laden 2% alginate (Pronova; FMC Biopolymer) at a cell density of 20×10^6^ MSCs/mL and allowed to gel for 30 min at 37°C. Constructs were cultured in a CM at 5% pO_2_ for 4 weeks to form the osseous component of the engineered phalanx. The osseous and chondral components were attached using a fibrin glue (same formulation as fibrin hydrogel described below) to form the tissue-engineered phalanx construct. Channeled tissue-engineered phalanx constructs were fabricated by generating a single axially aligned channel (Ø1.6 mm) within the MSC-seeded hydrogel immediately before the attachment of the osseous and chondral components. This axial channel was created by inserting a hypodermic needle into the engineered phalanx construct. The tissue-engineered alginate phalanx constructs (regular and channeled) were cultured for an additional week in a CM at 20% pO_2_ before subcutaneous implantation in nude mice.

### Fabrication and culture of tissue-engineered fibrin phalanx constructs

This study also explored if such engineered constructs could be generated using fibrin hydrogels, as opposed to alginate hydrogels. To this end, a two-part ABS phalanx mold was infiltrated with a 4% agarose solution and allowed to set. The resulting two-part 4% agarose mold was assembled and injected with an MSC-laden 50 mg/mL fibrinogen, 2.5 U/mL thrombin, 5,000 KIU/mL aprotinin, 17 mg/mL sodium chloride, and 20 mM CaCl_2_ solution at a cell density of 20×10^6^ MSCs/mL and allowed to gel for 30 min at 37°C. Constructs were cultured in a CM at 5% pO_2_ for 4 weeks to form a fibrin osseous component. The osseous and chondral components were attached using a fibrin sealant, maintained in a CM at 20% pO_2_ for an additional week, and implanted subcutaneously into nude mice.

### Fabrication and culture of tissue-engineered alginate TMJ condyle constructs

A two-part ABS mold was recapitulated from a 3D digitized image of a temporomandibular joint (TMJ) condyle, as described above. The two-part ABS mold was infiltrated with a 4% agarose/50 mM CaCl_2_ solution and allowed to set. The resulting two-part 4% agarose/50 mM CaCl_2_ mold was assembled and injected with MSC-laden 2% alginate at a cell density of 20×10^6^ MSCs/mL. TMJ condyle constructs were cultured in a CM at 5% pO_2_ for a period of 5 weeks, followed by culture in a hypertrophic medium consisting of hgDMEM GlutaMAX supplemented with 100 U/mL penicillin/streptomycin, 100 μg/mL sodium pyruvate, 40 μg/mL l-proline, 50 μg/mL l-ascorbic acid-2-phosphate, 4.7 μg/mL linoleic acid, 1.5 mg/mL bovine serum albumin, 1× insulin–transferrin–selenium, 1 nM dexamethasone, 2.5 μg/mL amphotericin B, 1 nM l-thyroxine, and 20 μg/mL β-glycerophosphate (both Sigma-Aldrich) at 20% pO_2_ for an additional 3 weeks.

### In vivo *subcutaneous implantation*

Tissue-engineered phalanx constructs were implanted subcutaneously into the back of nude mice (Balb/c; Harlan). Two subcutaneous pockets were made on either side of the spine, and a single construct was inserted into each pocket. Four constructs were implanted per group and constructs were harvested 8 weeks postimplantation. Mice were sacrificed by CO_2_ inhalation, and the animal protocol was reviewed and approved by the ethics committee of Trinity College Dublin.

### Histological and immunohistochemical analyses

Constructs were fixed in 4% paraformaldehyde, dehydrated in a graded series of ethanols, embedded in paraffin wax, sectioned at 8 μm, and affixed to microscope slides. Postimplantation constructs were decalcified in EDTA for up to 1 week. A cross-sectional slice of one postimplantation construct was embedded without decalcification treatment. The sections were stained with hematoxylin and eosin (H&E) to assess bone formation, picrosirius red to assess collagen distribution, 1% alizarin red to assess calcium accumulation, and aldehyde fuchsin/alcian blue to assess sGAG content. Collagen types I, II, and X were evaluated using a standard immunohistochemical technique; briefly, collagen I and II sections were treated with peroxidase, followed by treatment with chondroitinase ABC (Sigma-Aldrich) in a humidified environment at 37°C to enhance permeability of the extracellular matrix. Sections were incubated with goat serum to block nonspecific sites, and collagen type I (ab6308, 1:400; 1 mg/mL) or collagen type II (ab3092, 1:100; 1 mg/mL) primary antibodies (mouse monoclonal; Abcam) were applied overnight at 4°C, followed by incubation with the secondary antibody (anti-mouse IgG biotin conjugate, 1:200; 2.1 mg/mL) (Sigma-Aldrich) at room temperature for 1 h. Collagen type X sections were treated with peroxidase, pronase, and goat serum before incubation with the collagen type X primary antibody (ab49945, 1:100; 1.4 mg/mL) overnight at 4°C and application of the secondary antibody (ab49760, 1:100) at room temperature for 1 h. Thereafter, all sections were incubated with ABC reagent (Vectastain PK-400; Vector Labs) for 45 min. Finally, sections were developed with DAB peroxidase (Vector Labs) for 5 min. Positive and negative controls were included in the immunohistochemical staining protocols for each batch.

### Microcomputed tomography

Microcomputed tomography (μCT) scans were performed on constructs using a Scanco Medical 40 μCT system (Scanco Medical). Constructs were scanned in PBS, at a voxel resolution of 30 μm, a voltage of 70 kVp, and a current of 114 μA. A Gaussian filter (sigma=0.8, support=1) was used to suppress noise, and a global threshold corresponding to a density of 399.5 mg hydroxyapatite/cm^3^ was applied. 3D evaluation was carried out on the segmented images to reconstruct a 3D image. Evaluation was also carried out on center sections of the constructs corresponding to a thickness of 300 μm. Four constructs were analyzed per experimental group.

## Results

### *Development and* in vitro *culture of anatomically shaped cartilaginous grafts*

**FIG. 1.**
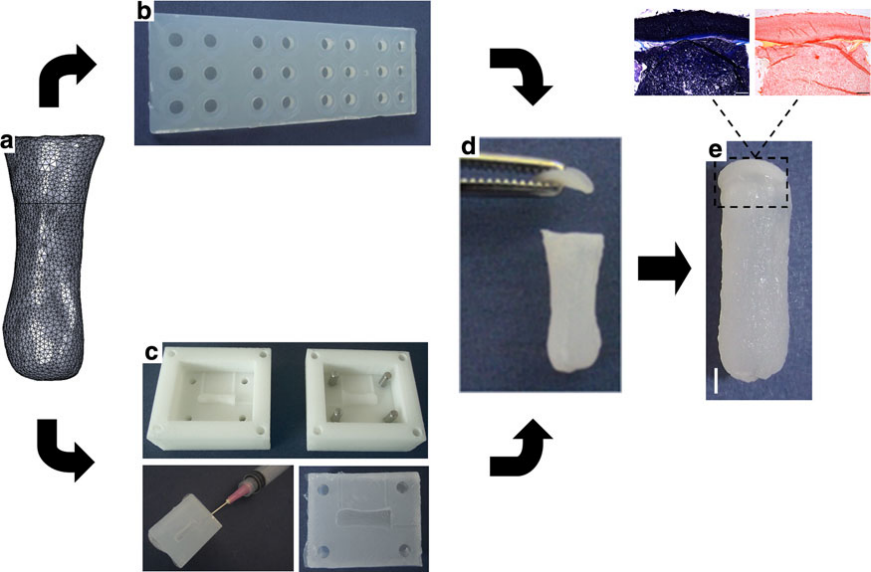
Anatomically shaped MSC-seeded alginate phalanx constructs were fabricated and cultured chondrogenically for 5 weeks. **(a)** 3D solid model of anatomically shaped phalanx construct. **(b)** Chondral layer molding system. Chondrocytes were self-assembled in cylindrical agarose wells. **(c)** Osseous component molding system. Clockwise from bottom; The two-part negative ABS mold, one half of the resultant positive 4% agarose/50 mM CaCl_2_ mold, assembly of the two-part agarose/CaCl_2_, and injection with MSC-laden alginate hydrogel. **(d)** Anatomically shaped constructs were formed by attaching a chondral layer (self-assembled chondrocytes, top) to an osseous component (MSC-laden alginate, bottom) using fibrin glue. **(e)** Macroscopic image of the anatomically shaped construct at the end of the in vitro culture period. Scale bar is 2 mm. Insets show the interface of the osseous and chondral components stained with aldehyde fuchsin/alcian blue (left) and picrosirius red (right). Inset scale bars are 500 μm. ABS, acrylonitrile–butadiene–styrene; MSC, mesenchymal stem cell.

The engineering of scaled-up, anatomically shaped, hypertrophic cartilaginous grafts to act as soft tissue templates for the regeneration of whole bones first required the fabrication of anatomical molds, which could be used to create MSC-laden hydrogels in the shape of different bones. To that end, the distal phalanx of a skeleton model was scanned using a 3D laser scanner and the resultant scans were rendered and meshed to reconstruct a 3D solid model (Fig. 1a). This model was sectioned in half and used to create a two-part reverse mold. These molds were filled with a 4% agarose/50 mM CaCl_2_ solution and, when set, were assembled and injected with MSC-laden 2% alginate to produce a template for the osseous or endochondral component of a tissue-engineered phalanx (Fig. 1c).

A further requirement of a functional engineered long bone is the development of a stable layer of articular cartilage at the articulating ends. To this end, primary chondrocytes were used to tissue engineer a chondral layer using a self-assembly approach (Fig. 1b), which was maintained in chondrocyte culture conditions for a period of 4 weeks. The osseous component and chondral layer were then attached (Fig. 1d) using a fibrin sealant and maintained in chondrocyte culture conditions for an additional week, resulting in a total *in vitro* culture period of 5 weeks. At the end of the 5-week *in vitro* culture period, the two components remained intact and generated a matrix rich in sGAG and collagen, as demonstrated by positive staining for aldehyde fuchsin/alcian blue and picrosirius red (Fig. 1e).

**FIG. 2.**
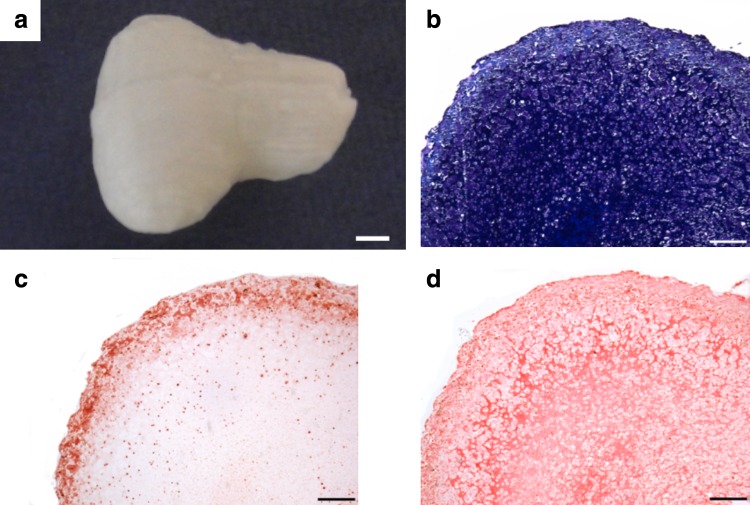
MSC-encapsulated alginate hydrogels were fabricated in the shape of the TMJ condyle. Constructs were maintained in chondrogenic culture conditions for a period of 5 weeks, followed by maintenance in hypertrophic culture conditions for an additional 3 weeks. **(a)** Macroscopic image of TMJ construct at week 8. Scale bar is 2 mm. **(b)** Aldehyde fuchsin/alcian blue, **(c)** alizarin red, and **(d)** picrosirius red staining of TMJ constructs at week 8 to assess sGAG, calcium, and collagen spatial accumulation, respectively. Scale bars are 500 μm. TMJ, temporomandibular joint.

To demonstrate the broad utility of this approach, the same methodology was also applied to engineer an MSC-laden alginate TMJ condyle. These constructs were cultured in a CM for 5 weeks, followed by culture in a hypertrophic medium for an additional 3 weeks (Fig. 2a). This led to the development a calcified cartilaginous construct *in vitro*, consisting of an inner cartilaginous matrix positively stained for aldehyde fuchsin/alcian blue and picrosirius red (Fig. 2b, d) and an outer calcified matrix positively stained for alizarin red (Fig. 2c).

### In vivo *development of tissue-engineered phalanx constructs*

**FIG. 3.**
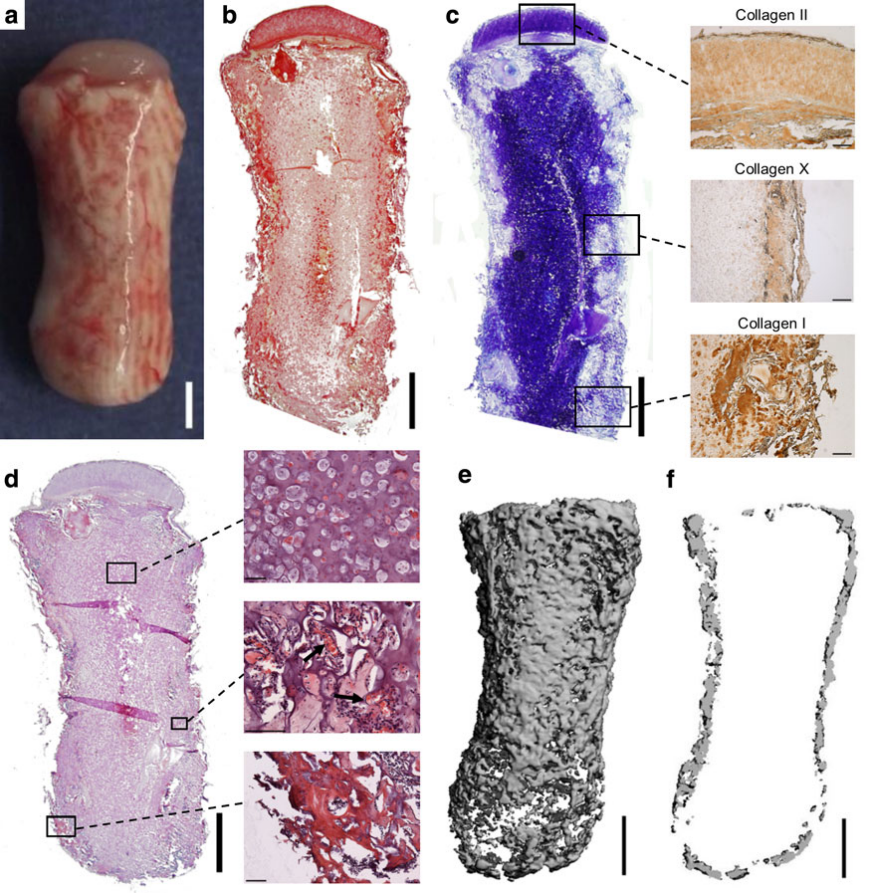
Anatomically shaped alginate phalanx constructs, cultured *in vitro* for 5 weeks, were implanted subcutaneously into nude mice and harvested 8 weeks postimplantation. **(a)** Macroscopic image of construct. **(b)** Picrosirius red staining of construct. **(c)** Aldehyde fuchsin/alcian blue staining of construct. Insets show collagen type II (top), collagen type X (center), and collagen type I (bottom) staining. **(d)** H&E staining of construct. **(e)** μCT image of whole construct. **(f)** μCT image of center section of construct corresponding to a thickness of 300 μm. Arrows in **(d)** indicate blood vessel structures. Main image scale bars are 2 mm. Inset scale bars in **(c)** are 250 μm. Inset scale bars in **(d)** are 100 μm. H&E, hematoxylin and eosin; μCT, microcomputed tomography.

Chondrogenically primed phalanx constructs were implanted subcutaneously into nude mice after 5 weeks of *in vitro* culture. After 8 weeks *in vivo*, the osseous component of the engineered phalanx was observed to undergo extensive calcification, with a clear vascular network developing, while the chondral layer remained intact and did not become vascularized (Fig. 3a). Constructs stained intensely for picrosirius red (indicating robust collagen accumulation) in the chondral layer and also around the periphery of the osseous component (Fig. 3b). In addition, the chondral layer stained positive for cartilage-specific extracellular matrix components—sGAGs and collagen type II (Fig. 3c; top inset)—and negative for the hypertrophic marker, collagen type X (data not shown). The calcified tissue around the periphery of the osseous component appeared to be bone forming through endochondral ossification, as evident by collagen type X immunostaining (Fig. 3c; middle inset), collagen type I immunostaining (Fig. 3c; bottom inset), and H&E staining, indicating the presence of bone-like tissue (Fig. 3d; bottom inset). Furthermore, a reduction in aldehyde fuchsin/alcian blue staining was observed in this region, indicating the transition from cartilage into bone, which was followed by infiltration of blood vessel structures (Fig. 3d; middle inset). Central regions in the osseous component appeared to retain the morphological characteristics of cartilage (Fig. 3d; top inset). μCT imaging confirmed the development of a heavily calcified outer matrix in the osseous component (Fig. 3e), with the central regions remaining uncalcified (Fig. 3f). No evidence of calcification in the chondral layer was apparent in μCT scans.

**FIG. 4.**
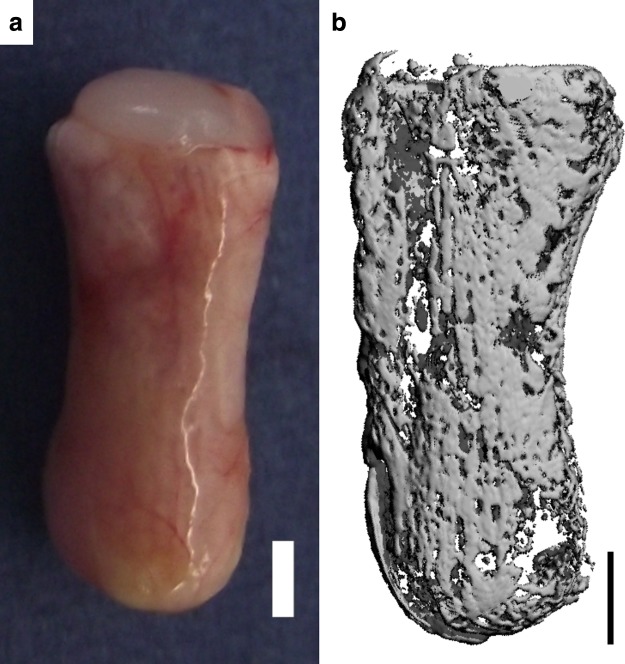
Anatomically shaped constructs consisting of an osseous component (MSC-encapsulated fibrin) and a chondral layer (self-assembled chondrocytes) were cultured for 5 weeks *in vitro* before subcutaneous implantation in nude mice for a period of 8 weeks. **(a)** Macroscopic image of the fibrin phalanx construct postimplantation. **(b)** μCT image of the fibrin phalanx construct postimplantation. Scale bars are 2 mm.

Anatomically shaped phalanx constructs consisting of an MSC-laden fibrin osseous component and a self-assembled chondrocyte chondral layer were also evaluated *in vivo.* Eight weeks postimplantation, such constructs also showed evidence of a vascular network surrounding a heavily calcified osseous component, with the chondral layer remaining intact (Fig. 4), confirming that the molding system developed in this study can be leveraged to tissue engineer anatomically shaped constructs using different hydrogels.

### Modifying the architecture of tissue-engineered phalanx constructs to accelerate bone formation

**FIG. 5.**
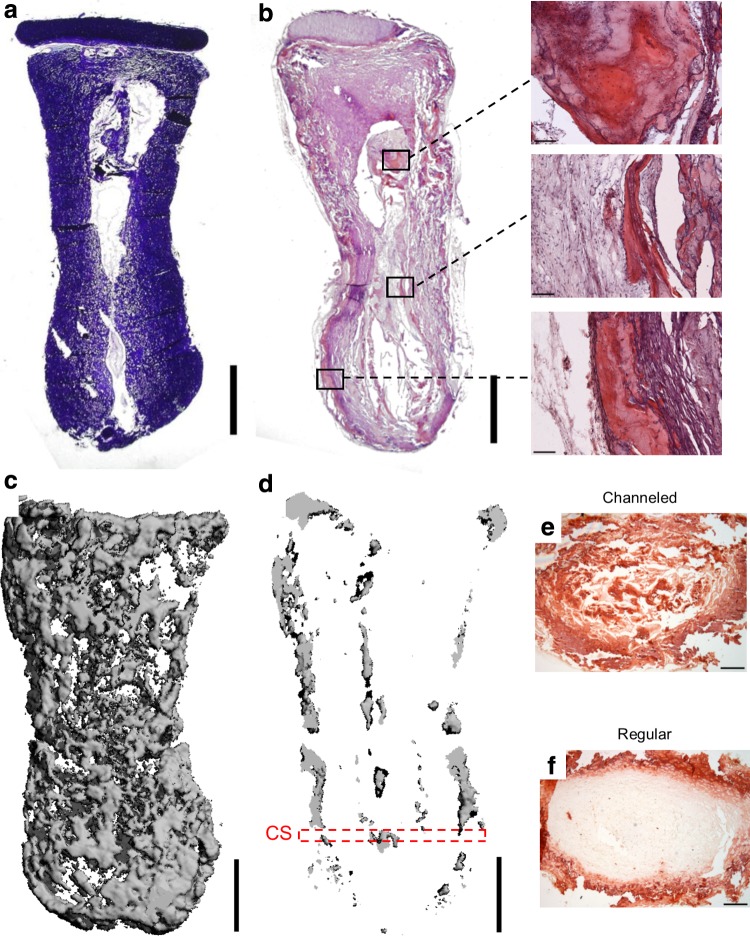
Before the attachment of the chondral layer, a single channel (Ø1.6 mm) was cored into the osseous component of the alginate phalanx construct. After 5 weeks of *in vitro* culture, constructs were subcutaneously implanted into nude mice for a period of 8 weeks. **(a)** Aldehyde fuchsin/alcian blue staining of channeled phalanx construct preimplantation. **(b)** H&E staining of channeled phalanx construct postimplantation. **(c)** μCT image of whole channeled construct. **(d)** μCT image of the center section of the channeled construct postimplantation corresponding to a thickness of 300 μm. **(e)** Alizarin red staining of the cross section of a channeled construct postimplantation. **(f)** Alizarin red staining of the cross section of a regular nonchanneled construct postimplantation. Cross sections were taken from the region CS, as illustrated in **(d)**. Scale bars in **(a–d)** are 2 mm. *Inset* scale bars in **(b)** are 100 μm. Scale bars in **(e–f)** are 500 μm.

**FIG. 6.**
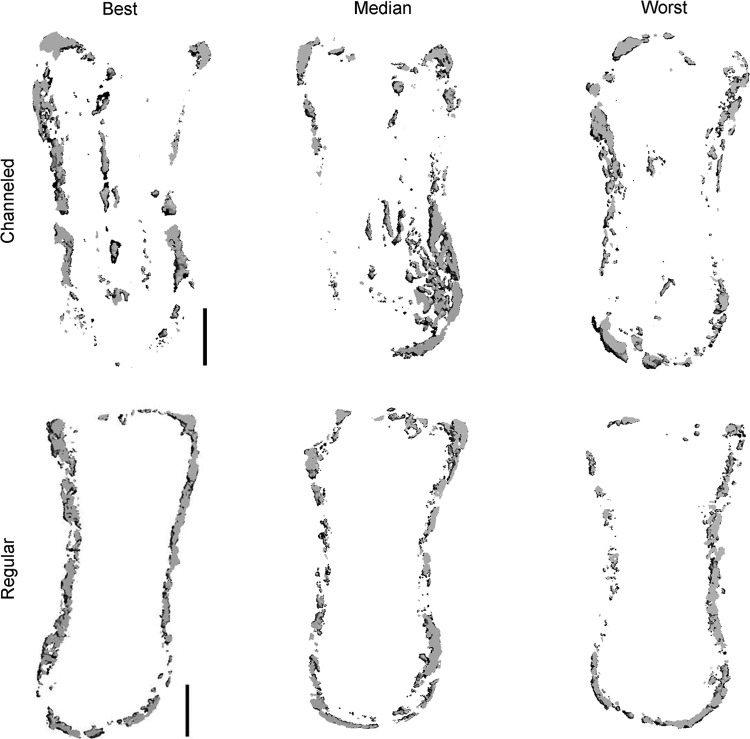
Best–Median–Worst graphs showing the variance in mineralization within channeled and regular alginate phalanx construct groups postimplantation. μCT images show center sections corresponding to thicknesses of 300 μm. Scale bars are 2 mm and are consistent across groups.

After 8 weeks *in vivo*, bone formation was limited to the periphery of the engineered phalanx (Fig. 3). In an attempt to accelerate bone formation throughout the tissue-engineered phalanx construct, we next modulated the architecture of the graft in an attempt to facilitate infiltration of host cells and vasculature. A single axially aligned cylindrical channel (Ø1.6 mm) was inserted into the longitudinal axis of the alginate osseous component of the construct before the attachment of the chondral layer (Fig. 5a). The channel remained partially patent for the duration of the *in vivo* study. H&E staining of the construct postimplantation demonstrated the formation of bone around the periphery and also in central regions adjacent to the channel (Fig. 5b). μCT imaging also demonstrated bone formation in peripheral and central regions of channeled constructs (Fig. 5c, d). Alizarin red staining of cross sections confirmed the enhancement of calcification in the center of channeled constructs (Fig. 5e) compared with regular nonchanneled constructs (Fig. 5f). μCT analysis (Fig. 6), however, revealed no significant difference in total bone density between channeled and regular nonchanneled constructs (channeled, 158.7±30.2 mg hydroxyapatite/cm^3^; regular, 162.3±13.3 mg hydroxyapatite/cm^3^).

## Discussion

This study examined the use of engineered anatomically shaped cartilaginous grafts for regenerating whole bones through endochondral ossification. Using digitized images of a range of different bones, it was possible to fabricate anatomic agarose molds, which, when filled with an MSC-laden hydrogel, recapitulated the shape of the human distal phalanx and TMJ condyle. The MSC-seeded alginate constructs could be cultured *in vitro* to generate a cartilaginous matrix, which stained homogenously for sGAG and collagen, surrounded by a peripheral layer of calcified tissue. Chondrogenically primed tissue-engineered phalanx constructs, consisting of an MSC-laden alginate hydrogel with an overlapping layer of articular cartilage generated by chondrocyte self-assembly, were found to undergo spatially regulated endochondral ossification *in vivo* with the chondral layer retaining its stable chondrogenic phenotype and the osseous component proceeding along the endochondral pathway and forming bone around its periphery. Modifying the architecture of the tissue-engineered phalanx construct, by inserting a single axially aligned channel into the alginate hydrogel before implantation, augmented the spatial distribution of bone formation within the hydrogel, leading to the development of a more homogenous osseous component. While this proof-of-principal study is limited to distal phalanx tissue engineering, we believe this endochondral strategy could be scaled to treat other conditions beyond phalanx regeneration.

A well-documented challenge with the scaling up of engineered tissues is the associated issue of nutrient limitation and waste removal. Recent work utilizing MSC-seeded collagen scaffolds for endochondral bone tissue engineering reported the *in vitro* development of a core region devoid of cells and matrix.^24^ A number of novel strategies have been implemented to ensure adequate nutrient delivery to and waste removal from engineered tissues, such as the application of dynamic culture conditions.^30–33^ However, the large phalanx and TMJ constructs engineered in this study showed homogenous cartilaginous matrix deposition after *in vitro* priming in free-swelling conditions, demonstrating the benefit of utilizing hydrogels when scaling up cartilaginous constructs for endochondral bone tissue engineering applications. Hydrogels possess an inherent advantage over the use of preformed porous scaffolds for the engineering of homogenous tissues as cells can be homogenously encapsulated within hydrogels. Furthermore, cells are limited in their capacity to migrate to regions of enhanced nutrient availability in the outer regions of the construct, thus preventing peripheral cell and tissue growth and their associated effects on nutrient transport. In spite of this, it should be noted that inhomogeneous tissues do form in MSC-laden hydrogels of a significant scale,^34^ and perhaps, at such very large dimensions, additional strategies such as bioreactor culture may be beneficial.

As the osseous component of the tissue-engineered phalanx construct, the current study employed an alginate hydrogel, which degrades over time,^35^ encapsulated with chondrogenically primed bone marrow-derived MSCs capable of surviving the initial hypoxic conditions experienced by engineered tissues upon *in vivo* implantation.^17^ This hypertrophic precursor of the engineered phalanx underwent endochondral ossification *in vivo*, leading to the development of a heavily calcified outer bony tissue penetrated with vasculature. However, tissue in central regions of the osseous component remained as cartilage. The lack of calcification may be explained, at least in part, by the relatively large volumes of the constructs used in this endochondral strategy (calculated as 336.9±38.7 mm^3^). For comparison, a recent scaled-up endochondral bone tissue engineering study used cylindrical cartilaginous constructs of dimensions Ø8×2 mm, equivalent to volumes of 100 mm^3^,^24^ over three-fold lower than those used in the current study. Another explanation for the inhibition of bone formation in the center of the engineered tissue may be the slow degradation of the unmodified alginate hydrogel. Accelerating the degradation rate of alginate hydrogels through gamma irradiation has been shown to enhance bone regeneration.^36^ Inflammatory cytokines may also be harnessed to efficiently remodel engineered cartilaginous grafts into bone.^37^

An alternative approach for the engineering of a more homogenous osseous tissue is modifying the architecture of the scaffold. We have recently demonstrated that the incorporation of channeled arrays into cylindrical hydrogels facilitates vascularization and enhances calcification of engineered hypertrophic cartilaginous constructs *in vivo.*^38^ In the current study, a single axially aligned channel inserted into the MSC-seeded alginate hydrogel promoted bone formation in the central region of the engineered phalanx. It would appear therefore that optimization of scaffold or hydrogel architecture is a key design criterion in the scaling up of large anatomically shaped grafts for whole bone regeneration.

As previously noted, a further requirement of a functional engineered long bone is the development of a stable layer of articular cartilage at the articulating ends. We have previously demonstrated that it is possible to engineer osteochondral tissues by implanting chondrogenically primed bilayered constructs containing both chondrocytes and MSCs and spatially regulating endochondral ossification.^28^ Motivated by this finding, primary chondrocytes were used to tissue engineer a chondral layer using a self-assembly or scaffold-free approach,^39–44^ which retained a stable chondrogenic phenotype following implantation, forming a matrix consisting of sGAG and collagen type II and void of collagen type X. There are, however, difficulties associated with obtaining sufficient numbers of chondrocytes for the engineering of large cartilaginous layers.^45,46^ Previous studies have demonstrated, *in vitro*, a beneficial effect of coculturing a small number of chondrocytes with a larger number of MSCs.^47–50^ Future work in our laboratory will investigate if cocultures of chondrocytes and MSCs can be utilized to generate scaled-up stable cartilaginous grafts *in vivo.*

This study employed a subcutaneous environment to facilitate the development of an engineered cartilaginous construct into an endochondral bone tissue. From a translational perspective, this approach may also be adopted in the clinic, that is, using an ectopic environment as an *in vivo* bioreactor^51,52^ to allow maturation of an engineered tissue, with functional vasculature and marrow components,^24^ which can then be implanted into an orthotopic defect site. The alternative would be to forsake the ectopic transplantation and implant the engineered cartilaginous graft directly into the defect site, allowing endochondral ossification to occur orthotopically. This approach has been demonstrated using a coral scaffold, which would have an inherent advantage over a hydrogel in performing an immediate mechanical function, for the replacement of an avulsed phalanx.^5^ The relatively lower load-bearing environment of the upper limbs may allow for direct implantation of an engineered cartilaginous construct into a bone defect, although it may be more challenging if applied in a mechanically loaded defect site in the lower limb, which would require the engineered tissue to perform a more demanding biomechanical function. (Previous studies from our laboratory have shown that the equilibrium compressive modulus of cartilaginous tissues engineered using bone marrow-derived MSCs is typically less than 100 kPa,^33,53–55^ potentially limiting their use in mechanically challenging environments). Further studies using larger animal models are required to compare the efficacy of ectopic and orthotopic strategies in endochondral bone tissue engineering applications.

In conclusion, this work demonstrates the potential of utilizing anatomically shaped cartilaginous grafts for the tissue engineering of whole bones through endochondral ossification. An MSC-laden alginate hydrogel served as the osseous or endochondral component of an engineered phalanx construct, and a self-assembly strategy was used to engineer the overlapping articular cartilage layer using primary chondrocytes, as opposed to MSCs. The chondrogenically primed phalanx constructs were found to undergo spatially regulated endochondral ossification *in vivo*, with the osseous component engineered using bone marrow-derived MSCs proceeding along the endochondral pathway, and no evidence of calcification being observed in the integrated layer of self-assembled chondrocytes. Modifying the architecture of phalanx constructs, by inserting a single channel into the alginate hydrogel before implantation, accelerated bone formation in the center of the engineered construct and facilitated the development of a more homogenous osseous tissue.

## Abbreviations Used

ABS: acrylonitrile–butadiene–styrene
CM: chondrogenic medium
FBS: fetal bovine serum
H&E: hematoxylin and eosin
hgDMEM: high-glucose Dulbecco's modified Eagle's medium
MSC: mesenchymal stem cell
μCT: microcomputed tomography
PBS: phosphate-buffered saline
3D: three-dimensional
TMJ: temporomandibular joint
